# Impact of COVID-19 on pornography use: Evidence from big data analyses

**DOI:** 10.1371/journal.pone.0260386

**Published:** 2021-12-21

**Authors:** Way Kwok-Wai Lau, Lionel Ho-Man Ngan, Randolph Chun-Ho Chan, William Ka-Kei Wu, Benson Wui-Man Lau

**Affiliations:** 1 Department of Special Education and Counselling, The Education University of Hong Kong, Ting Kok, Hong Kong; 2 Department of Rehabilitation Sciences, The Hong Kong Polytechnic University, Hung Hom, Hong Kong; 3 Department of Anaesthesia and Intensive Care, Peter Hung Pain Research Institute and Li Ka Shing Institute of Health Sciences, The Chinese University of Hong Kong, Central Ave, Hong Kong; The University of Hong Kong, HONG KONG

## Abstract

**Introduction:**

Coronavirus disease 2019 (COVID-19) has led to radical changes in social distancing awareness and affected social relationships. Owing to large-scale lockdown, home quarantine and social distancing requirements, it was anticipated that sexual activities would be severely impacted. However, retrospective self-report studies showed that pornography use and autoerotism increased during the pandemic.

**Aim:**

This study used big-data databases available on the Internet to investigate factors that modulated pornography use during the pandemic.

**Methods:**

Daily relative search volume (RSV) data from Google Trends for the period from 24 February 2020 to 13 July 2020 were extracted. Pornhub traffic data were extracted from the Pornhub Insights website, for the period from 24 February 2020 to 13 July 2020. The parameter was defined as ‘percent change in traffic compared to an average day in 2019’. The number of daily new cases of COVID-19 was extracted from the database on Our World in Data.

**Outcome measures:**

The normality of the data was examined using the Shapiro-Wilk test. All variables included in this study were non-normally distributed. Therefore, non-parametric tests or parametric tests with bootstrapping were adopted where appropriate.

**Results:**

According to Google Trends, the RSV for ‘pornography’ increased after late March 2020, which is close to the date when the World Health Organization declared COVID-19 a global pandemic. The number of daily new cases of COVID-19 was positively correlated with the traffic of Pornhub, a popular pornography website, and the RSV for ‘pornography’. Moderation analysis demonstrated a significant main effect of daily new cases of COVID-19 and the RSV for ‘social distancing’ in predicting Pornhub traffic/RSV for ‘pornography’. Furthermore, the RSV for ‘social distancing’ significantly moderated the relationship between daily new cases and Pornhub traffic/RSV for ‘pornography’. A stronger COVID-pornography use association was observed with increased social distancing awareness.

**Conclusion:**

Increased pornography consumption during the pandemic was observed, and it was associated with the severity of the pandemic. Social distancing awareness could be a key factor influencing interest in and use of pornography. Further studies on the changes in sexual desire and birth-rate control are worthwhile because long-term public health may be affected by the changes in sexual behaviour during the pandemic.

## Introduction

Coronavirus disease 2019 (COVID-19) is an infectious disease caused by severe acute respiratory syndrome coronavirus 2 (SARS-CoV-2). According to early studies on the transmission of SARS-CoV-2, the virus is mainly spread through respiratory droplets; other potential transmission routes such as urine and faeces are under investigation [1, 2]. Although there is still no concrete evidence on the sexual transmission of SARS-CoV-2, sexual practices such as kissing, anal intercourse and analingus can increase the risk of SARS-CoV-2 transmission [3, 4] owing to the close interpersonal contact. On 11 March 2020, the World Health Organization (WHO) declared COVID-19 a global pandemic [5]. To minimise the risk of human-to-human transmission through direct interpersonal physical contact [6], governments of different countries issued quarantine and social distancing guidelines. Because the disease can be transmitted through respiratory droplets and short-distance interhuman contact, social distancing is considered an effective prevention measure. Nevertheless, these measures have dramatically changed interpersonal, social and potentially sexual relationships [6].

It is postulated that the sexuality of the general public can be affected by the transmission of SARS-CoV-2. For example, survey studies conducted in different countries have reported a decreased frequency of sexual interaction and risky sexual behaviours among heterosexual and homosexual adults [7–9]. Various studies have presented a few unambiguous findings such as the increased use of pornography. Pornhub, one of the largest providers of free online pornographic videos, has reported increased traffic after the outbreak of the disease compared with the average traffic in 2019 [10]. A study that used Google Trends, a tool that indicates the interest of the public in a particular search term, reported that the relative search volume (RSV) of Pornhub increased after the beginning of the pandemic. During this period, Pornhub showed the highest increase of interest worldwide compared with other popular porn websites such as Porn, NXX, xVideos and xHamster [11]. The same study concluded that Pornhub was the most popular porn website during the pandemic [11], making it the ideal marker for pornography consumption. It was also reported that the frequency of autoerotism, pornography consumption and use of phone sex increased [12]. Because large-scale lockdowns are common during the pandemic, pornography use and autoerotism may become important channels through which to express sexuality, thus enabling the fulfilment of sexual desires during this period that emphasises social distancing.

Early studies on sexual activities, including autoerotism, during the pandemic used online surveys, with possible self-reporting bias. In addition, customised, non-standardised questionnaires and questions were often used, which may increase difficulties in comparing results among studies and potentially lead to invalid conclusions. Furthermore, it is difficult to explore the possible underlying factors that may mediate or moderate sexual activities. Objective data on the impact of COVID-19 on sex are therefore lacking [13].

In light of the above-mentioned research gaps, this study aims to explore the change in pornography use with big data databases available on the Internet. The objective of this study is to explore associations between the emerging situation of COVID-19, social distancing awareness and pornography use. In addition, we hypothesise that social distancing awareness can moderate the association between the severity of the COVID-19 infection and pornography consumption during the pandemic. Our findings add to a growing body of the literature and improve our understanding of the relationship between the pandemic and pornography use by providing objective data from the Internet to analyse the changes in pornography use during the pandemic.

## Methods

### Data extraction

Data used in this study were extracted from three different sources, namely, ‘Google Trends’, ‘Pornhub Insights’ [14] and ‘Our World in Data’ [15], in July 2020. We extracted daily RSV data for the period from 24 February 2020 to 13 July 2020. The search terms included ‘COVID’, ‘social distancing’ and ‘pornography’. The extracted data were denoted as RSV. Pornhub traffic data were extracted from the website ‘Pornhub Insights’ [14]. The parameter was defined as ‘percent change in traffic compared to an average day in 2019’. The number of daily new cases of COVID-19 globally was extracted from the database on Our World in Data [15].

### Statistical analysis

The normality of the data was examined using the Shapiro-Wilk test. All variables included in this study were non-normally distributed. Therefore, non-parametric tests or parametric tests with bootstrapping were adopted where appropriate.

The associations between the RSVs for ‘social distancing’, ‘COVID’ and ‘pornography’, the number of daily new cases, and the Pornhub traffic rate were analysed using Spearman-rho correlation analyses. Ten comparisons were conducted; thus, *P* < .005 (0.05/10) is defined as statistically significant after Bonferroni correction.

To determine the role of social distancing awareness on the association between the number of daily new cases and pornography use, moderation analyses were performed using model 1 in the PROCESS macro for Statistical Package for Social Science (SPSS) that was developed by Hayes [16]. The PROCESS macro is based on ordinary least squares regression and adopts a nonparametric bootstrapping procedure (5000 bootstrapped samples in this study), which provides a bias-corrected confidence interval (CI) for effect size inference [17]. Statistical significance is established if the 95% CI does not cross zero [18]. The number of daily new cases (predictors) and the RSV for ‘social distancing’ (moderators) were mean-centred. The mean-centred variables and their interaction term were entered into the linear regression model for predicting the traffic rate of Pornhub or the RSV for ‘pornography’. The binary variable, i.e. free promotion period of Pornhub (yes/no), was entered into the regression model as a control variable.

All statistical analyses were conducted using SPSS v24 (IBM). A *P*-value < 0.05 is considered statistically significant unless otherwise specified.

## Results

### Positive association between the number of daily new COVID-19 cases and Pornhub traffic

**Fig 1:**
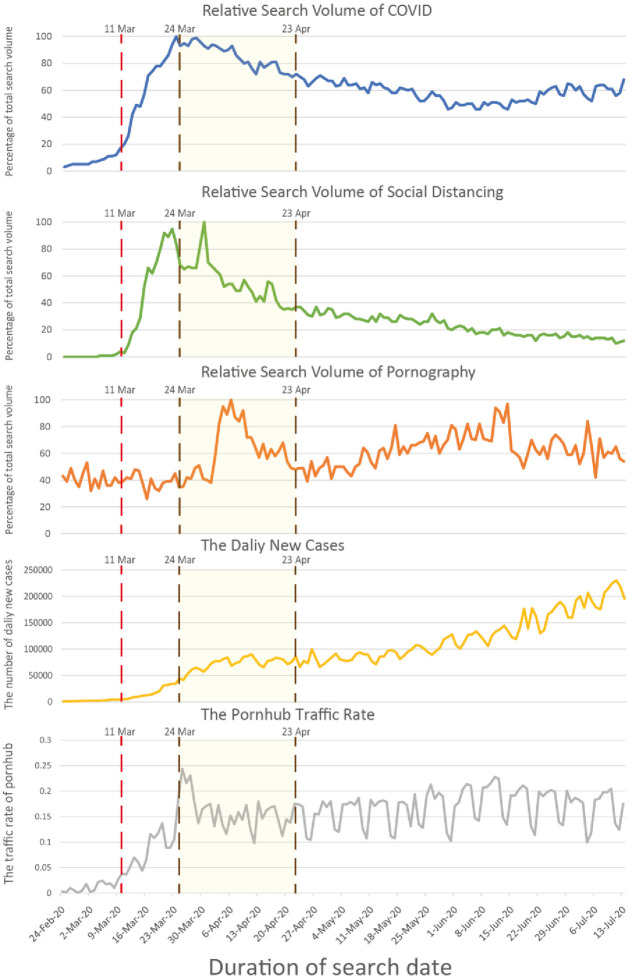
Relative search volume of ‘COVID’, ‘Social distancing’ and ‘Pornography’ from Google Trend(s); the Pornhub traffic rate and COVID-19 daily new cases. Note: On Mar 11, 2020, the World Health Organization (WHO) announced COVID-19 to be a global pandemic. Free promotion period of Pornhub (Premium) from Mar 24 –Apr 23, 2020.

In total, 141 data points were collected from 24 February to 13 July 2020 through the sources described above. The RSVs for the terms ‘COVID’, ‘social distancing’ and ‘pornography’, the Pornhub traffic rate and COVID-19 daily new cases are presented in Fig 1. A notable increase in the RSVs for ‘COVID’ and ‘social distancing’ was observed in early March and that for ‘pornography’ was observed in late March. A strong positive association between the number of daily new cases and the Pornhub traffic rate was observed at the initial phase of the pandemic when the number of new cases was approximately 50,000 globally. The growth curve of the traffic rate exhibited a plateau when the number of new cases exceeded 50,000. The free promotion period of Pornhub (from 25 to 27 March 2020) also had a positive effect on the Pornhub traffic rate. S1 Fig shows the scatter plot of the Pornhub traffic data and daily new cases of COVID-19.

Associations among RSVs for ‘social distancing’, ‘COVID’ and ‘pornography’, the daily new cases and the Pornhub traffic rate from 24 February to 13 July 2020.

Correlation coefficients among the variables (RSVs, number of daily new cases and traffic data) by Spearman’s rho are shown in Table 1. The number of daily new cases of COVID-19 was positively related to the Pornhub traffic (rho = 0.562, *P* < .001) and the RSV for ‘pornography’ (rho = 0.698, *P* < .001) and negatively related to the RSV for ‘social distancing’ (rho = -0.284, *P* < .001). As expected, the RSV for ‘pornography’ was significantly correlated with the Pornhub traffic (rho = 0.343, *P* < .001). There was a positive, strong association between the RSVs of ‘social distancing’ and ‘COVID’ (rho = 0.849, *P* < .001).

**Table 1:** Spearman’s rho correlation among studied variables.

	1	2	3	4
1. RSV for ‘Social Distancing’	---			
2. RSV for ‘COVID’	**0.849 (.001)**			
3. RSV for ‘Pornography’	-0.085 (.31)	-0.082 (.34)		
4. Daily new cases of COVID-19	**-0.284 (.001)**	-0.101 (.23)	**0.698 (.001)**	
5. Traffic of Pornhub	0.144 (.09)	0.229 (.006)	**0.343 (.001)**	**0.562 (.001)**

Numerical data represent the Spearman’s rho values and the corresponding *P*-values in brackets. *P* < .005 is defined as statistically significant after Bonferroni correction. Significant correlations are written in bold text.

### Moderating role of social distancing awareness in daily new cases-pornography use association

**Fig 2:**
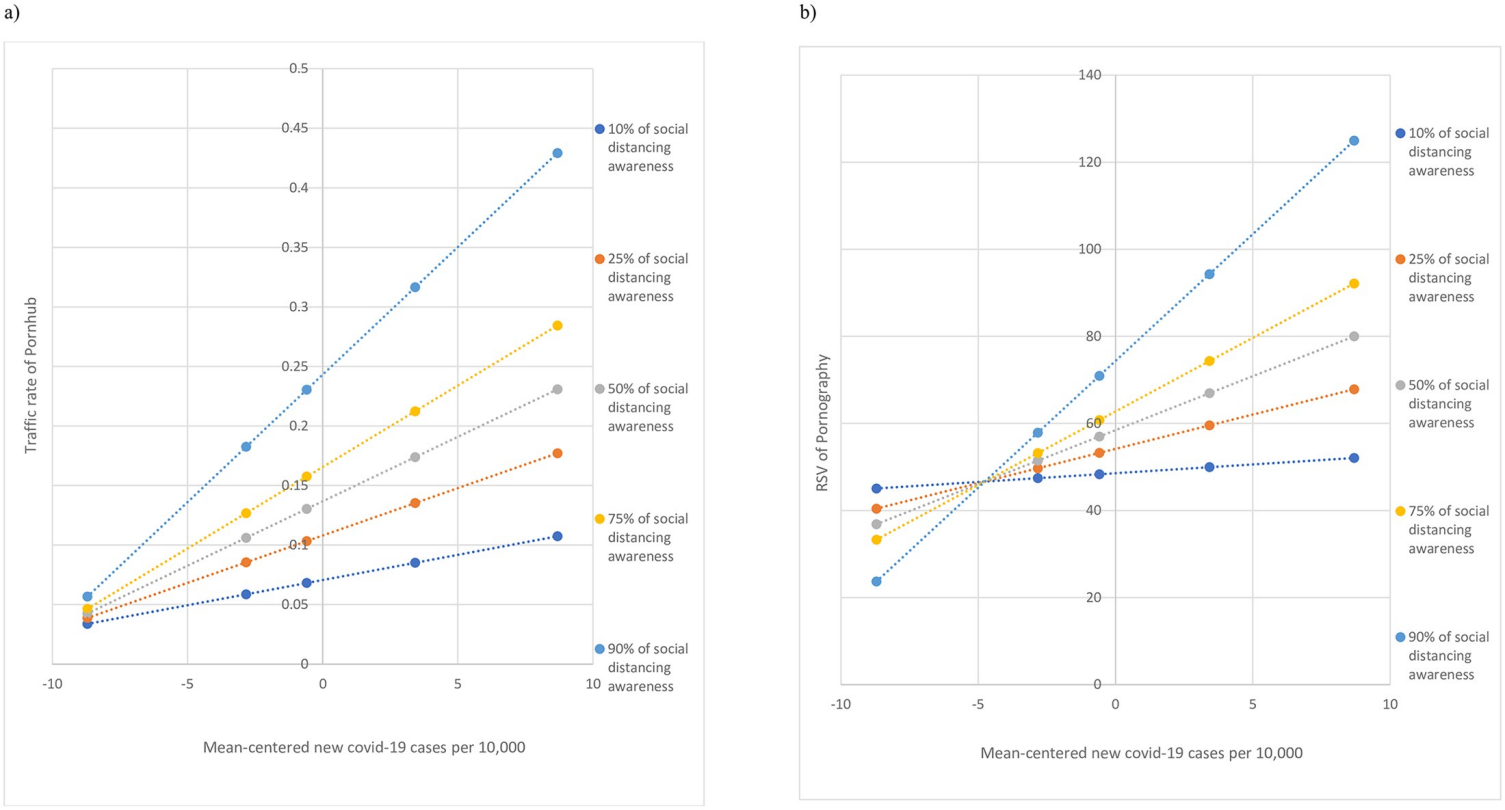
a: Moderation effect of the awareness of social distancing on the relationship between covid-19 new cases and traffic rate of Pornhub. b: Moderation effect of the awareness of social distancing on the relationship between covid-19 new cases and RSV of ‘pornography’.

The regression model for predicting Pornhub traffic rate using the number of daily new cases, the RSV for ‘social distancing’ and their interaction term was significant after we controlled for the effect of free access to Pornhub premium service [*F*_*(4*, *136)*_ = 52.571, *P* < .001], which explained 60.73% variance of Pornhub traffic rate. The number of daily new cases (10,000 per unit) (*β* = 0.012, *SE* = 0.001, 95% *CI* = 0.010–0.015, *P* < .001), the RSV for ‘social distancing’ (*β* = 0.003, *SE* = 0.001, 95% *CI* = 0.002–0.004, *P* < .001) and their interaction term (*β* = 0.0003, *SE* = 0.0001, 95% *CI* = 0.0001–0.0004, *P* < .001) were significant predictors of Pornhub traffic rate. The addition of the interaction term significantly increased the *R*^*2*^ of the model [*R*^*2*^-change = 0.043, *F*_*(1*,*136)*_ = 14.991, *P* < .001]. The significant interaction term supported the moderating role of RSV for ‘social distancing’ on the relationship between the number of daily new cases and the Pornhub traffic rate in which an increase in the RSV for ‘social distancing’ enhanced such an association (Fig 2a).

To further confirm our findings, the same moderation analysis was performed by replacing the dependent variable with the RSV for ‘pornography’. The RSV for ‘pornography’ indicated the interest in pornography, which may include the surfing of pornography websites other than Pornhub. The regression model for predicting the RSV for ‘pornography’ was significant [*F*_*(4*, *136)*_ = 29.828, *P* < .001], which explained 46.73% of variance of the RSV for ‘pornography’. The number of daily new cases (10,000 per unit) (*β* = 2.907, *SE* = 0.368, 95% *CI* = 2.179–3.635, *P* < .001), the RSV for ‘social distancing’ (*β* = 0.430, *SE* = 0.133, 95% *CI* = 0.167–0.693, *P* = .002) and their interaction term (*β* = 0.090, *SE* = 0.021, 95% *CI* = 0.050–0.131, *P* < .001) were significant predictors of the RSV for ‘pornography’. The addition of the interaction term significantly increased the *R*^*2*^ of the model [*R*^*2*^-change = 0.075, *F*_*(1*,*136)*_ = 19.181, *P* < .001] (Fig 2b). The increase in the RSV for ‘social distancing’ enhanced the association between the number of daily new cases and the RSV for ‘pornography’. Interestingly, the moderation effect of the RSV for ‘social distancing’ at the first 10^th^ percentile was non-significant (*P* = 0.17).

## Discussion

According to Pornhub Insights, an increase in traffic on Pornhub.com was observed from early March 2020. This increase in traffic peaked (+24.4% compared to average daily traffic in 2019) on 25 March 2020, and it was maintained at a level from +9.8% to +22.8% until June 2020 [14]. Our results showed that the increase in Pornhub traffic was significantly associated with the daily new cases of COVID-19, and the traffic could be predicted by the daily new cases. These findings imply that the trend of pornography use may be consistent with the development of the pandemic. The findings agree with previous studies in which the frequency of autoerotism and pornography use was reported to increase during the pandemic [12, 19].

The increase in pornography consumption could represent a maladaptive coping strategy toward stressors, i.e. fear and feelings of powerlessness triggered by the pandemic. Masaeli and Farhadi suggested that long-term increased pornography consumption could result in Internet addiction, which could negatively impact an individual’s psychosocial and/or physical well-being [20]. Consistent with this idea, Marchi and colleagues proposed that problematic pornography consumption, i.e. high-frequency consumption that can cause possible side effects in legal, psychological and physical health, might be considered a type of addiction requiring clinical attention [21]. Awan and colleagues further reviewed the plausible neuropsychological mechanisms of pornography addition that involve the inhibitory and reward systems to explain this phenomenon [22]. For example, more frequent consumption of pornographic content was associated with smaller right caudate volume, suggesting a constant stimulation of the reward system or a neuroplastic change that further enhances the pleasant feeling of consuming pornographic content [23]. Furthermore, problematic Internet behaviours were found to be negatively correlated and positively correlated with the brain activities in default mode network and inhibitory control network, respectively, similar to other substance and behavioural addictions [24]. Our findings indicate the potential role of social distancing awareness as a positive moderator of the relationship between daily new cases of COVID-19 and pornography use, which supports the notion that the current pandemic has increased the odds in the development of pornography addiction that requires clinical attention.

More than 40% of respondents in a survey conducted in Italy reported an increase in sexual desire during quarantine [12]. Similarly, another study conducted in Southeast Asian countries reported elevated libido [25]. However, the increase in sexual desire was not associated with an increase in sexual intercourse [12]. Instead, autoerotism and pornography use may have replaced actual sexual intercourse to fulfil sexual needs. These findings support the idea that pornography is an example of supranormal stimuli [26]. Supranormal stimulus in the context of pornography refers to the greater stimulation of the reward system by pornography materials, i.e. porn videos or photos, than by actual sexual intercourse [27]. Overconsumption of pornography can cause loss of interest in actual sexual intercourse because individuals cannot achieve the same responses in real life as those they achieve from pornography [28], which may negatively impact their mental well-being. Further empirical studies are required to investigate this area.

There are many reasons why there is an increase in pornography consumption during the pandemic and lockdowns. According to the ‘Triple A Engine’ hypothesis suggested by Cooper [29], the accessible, affordable and anonymous nature of the Internet makes online consumption of pornography relatively easy. The key characteristics of the three ‘A’s are easy accessibility, provision of free pornography services and privacy of an individual’s identity. These characteristics of the internet allow individuals to easily express their sexualities online and provide sexual outlets for them to meet their sexual needs and reduce loneliness during lockdowns. Stressors related to the pandemic and increased sexual desire may encourage people to engage in pornography use. Negative emotions and psychological effects including anger, confusion, anxiety and depression were reported to be caused by quarantines, fear of infection, boredom and inadequate support from others [30]. Another possible reason for increased pornography use may be ‘forced abstinence’ [31]. It is reported that when the servers for a popular video game crashed, Pornhub traffic increased and the keyword search for pornographic videos with the game’s name increased by 60%. This observation suggests that the gamers may have developed a compensatory behaviour (viewing pornography) during the period of ‘forced abstinence (cannot access game servers)’. In the current pandemic situation, the increased pornography consumption may be viewed as a compensatory behaviour that compensates for the sexual needs of ‘abstinence’ from real-life sexual activities. Interestingly, the increased pornography consumption may also be attributed to opinions from professionals [32]. During lockdowns, staying at home and using pornography were seen less as problematic and risky behaviour, and it was viewed as a desirable behaviour to prevent the transmission of COVID-19. Pornography and masturbation were viewed as low-risk sexual outlets to ensure safety and maintain social distance among people.

A significant reduction in female sexual functioning and quality of life was observed during the pandemic [33]. Furthermore, the presence of anxiety and risk perception of COVID-19 were observed to be negatively associated with the frequency of sexual activity [34]. As wellbeing is generally considered to be positively associated with the frequency of sexual intercourse [35], a decrease in the frequency of sexual intercourse may indicate a decrease in mental wellbeing, even when autoerotism and pornography are used as substitutes for sexual intercourse. It was hypothesised that pornography consumption is used as a coping mechanism to deal with affection deprivation [36]. However, its long-term effect on mental health is still controversial. It was observed that pornography consumption is inversely related to relationship satisfaction and closeness, and it is positively related to loneliness and depression. This finding agrees with our observation that lockdowns are positively associated with increased pornography use. In different hypotheses, decreased sexual satisfaction owing to unrealistic expectations of sexual partners and performance may be caused by long-term pornography use [37]. It was suggested that this effect of pornography use is associated with a changed brain reward circuit in response to pornography, as the real-life situation may not be as ideal, novel and attractive as online pornography [38]. The disappointment resulting from comparing real-life to online images may cause dissatisfaction in sex.

Increased pornography consumption was associated with lower commitment to a romantic partner [39] and more frequent flirting with an extradyadic partner. However, because association does not imply causation, the association between pornography consumption and infidelity may not imply the long-term consequence of pornography consumption. Similarly, it was hypothesised that pornography can potentially cause sexual dysfunction, but the association between pornography consumption and sexual dysfunction was found to be weak. Therefore, pornography consumption appears not to be a major risk factor of sexual dysfunction [40].

During the pandemic, the public has increased awareness and/or concerns of ‘social distancing’, which is indicated by the increased RSV and the strong positive correlation between the RSVs for ‘Social Distancing’ and ‘COVID’. Therefore, we hypothesised that social distancing awareness would moderate the association between the daily number of new COVID-19 cases and pornography consumption. This hypothesis is supported by the moderation analysis in this study. The results showed that the positive association between the daily number of new COVID-19 cases and pornography consumption was stronger during periods of increased social distancing awareness. Interestingly, when the RSV for ‘social distancing’ was at a relatively low level (below 10^th^ percentile), the number of daily new cases was not associated with the RSV for ‘pornography’. One explanation for this finding is that pornography consumption increased with the daily new cases of COVID-19 only when the general public became aware of social distancing, suggesting that social distancing awareness could be a key factor affecting the display of autoerotism. However, we cannot rule out the possibility that quarantine and lockdown policies indifferent countries during the pandemic could have resulted in increased pornography consumption [11].

The search volume for social distancing increased rapidly from early March and peaked at the end of March 2020, which is temporally close to the date when WHO declared COVID-19 a global pandemic. The increased concern about social distancing could imply the increased awareness of transmission routes of COVID-19. The idea that sexual intercourse can transmit SARS-CoV-2, may decrease sexual activity among non-cohabitating partners [32]. However, sexual activity among non-cohabitating partners may also be reduced because of quarantines. Meanwhile, sexual activity among non-infected cohabitating partners is considered safe [4], although the frequency of sexual intercourse may be suppressed by factors including the presence of children at home (privacy issue) and negative psychological factors and mood [13]. With increased social distancing awareness, it is understandable that sexual intercourse may be avoided and substituted by autoerotism to fulfil sexual desires. Other confounding factors such as quarantines, privacy issues at home and negative moods could also reduce sexual activity among non-cohabitating and cohabitating partners, resulting in increased consumption of pornography for compensation. Future studies that control these factors can further confirm our findings.

According to findings on the transmission of COVID-19, the Italian Society of Andrology (ISA) provided guidelines on sexual practices to minimise the risk of infection [6]. They suggested possible safe sexual practices including intercourse between uninfected partners, use of sex toys and masturbation; however, they advised against practices such as kissing, analingus and sexual contact with partners outside of the home. The Spanish Association of Sexuality and Mental Health provided recommendations similar to those of ISA, with additional suggestions on avoiding sexual activities during quarantine and substituting intercourse with masturbatory or virtual sexual activity [41]. Our findings indicate that these guidelines may have influenced the increase in social distancing awareness, thus facilitating the positive relationship between pandemic severity and pornography consumption.

A future research question worth exploring is why sexual desire is increased during the pandemic. Loneliness and negative emotions may increase the urge for intimate relationships to reduce stress and improve physical health [42]. Moreover, increased pornography consumption could be attributed to the increased use of the Internet during lockdowns [43]. A study conducted on elderlies aged 60 years or more observed a significant increase in Internet use among the subjects; Internet use and online communication increased by 64.1% and 41.7%, respectively. It was suggested that the increase in Internet use led to an increase in the consumption of online pornography [44]. According to the findings of Awan et al. [22], the peak increase in Pornhub traffic often coincides with or appears soon after the beginning of a lockdown. From the data available on Pornhub Insights, this phenomenon can be observed in countries such as Italy, France, Spain, the US, Brazil, UK and India.

Although pornography use or autoerotism may satisfy solitary sexual desires, the lack of intimate relationships and the unfulfillment of dyadic sexual desire cannot be addressed through this means, and thus sexual pleasure and satisfaction may not be achieved even with increased pornography use [12]. Furthermore, there are concerns about mental health problems due to increased pornography consumption [22]. It is suggested that online pornography consumption can cause addiction owing to multiple reasons (accessibility, affordability and anonymity) [45], and its detrimental effects may be similar to those of Internet addiction and behavioural addiction, which are characterised by excessive preoccupations, urges or behaviours regarding the consumption. These effects may cause distress at the individual level, failure to reduce the consumption and other adverse psychological consequences. In addition, the supranormal nature of pornography may increase the difficulty in experiencing sexual satisfaction in real life, which would also distress the affected individuals.

As the pandemic is ongoing, its full impact on sexuality is yet to be seen. In this study, we found that the daily new cases of COVID-19 globally and social distancing awareness are associated with increased pornography consumption during the pandemic. We attempted to present a global picture regarding the associations between COVID-19 new cases, social distancing awareness and pornography consumption. Such associations can vary in individual countries because the timeline of the outbreak, number of new cases and lockdown policies are different across countries. Future studies are required to further investigate the cross-countries effect on these associations. As sexual behaviour is closely related to mental and sexual health, follow-up research is required to understand the influence of the changes on these areas, which may be the secondary impacts of COVID.

## Supporting information

S1 Fig Scatter plots showing correlation between Pornhub traffic and new daily average Covid19 cases. Traffic of Pornhub, percentage change compared to average day before COVID-19.
(PDF)
