# Supplementary material for: Impact of COVID-19 on pornography use: Evidence from big data analyses
Source: PLoS One. 2021 Dec 21;16(12):e0260386. doi: 10.1371/journal.pone.0260386 (PMC8691607; doi:10.1371/journal.pone.0260386)

Supplementary Material

S1 Fig. Scatter plots showing correlation between Pornhub Traffic and New Daily average Covid19 cases.

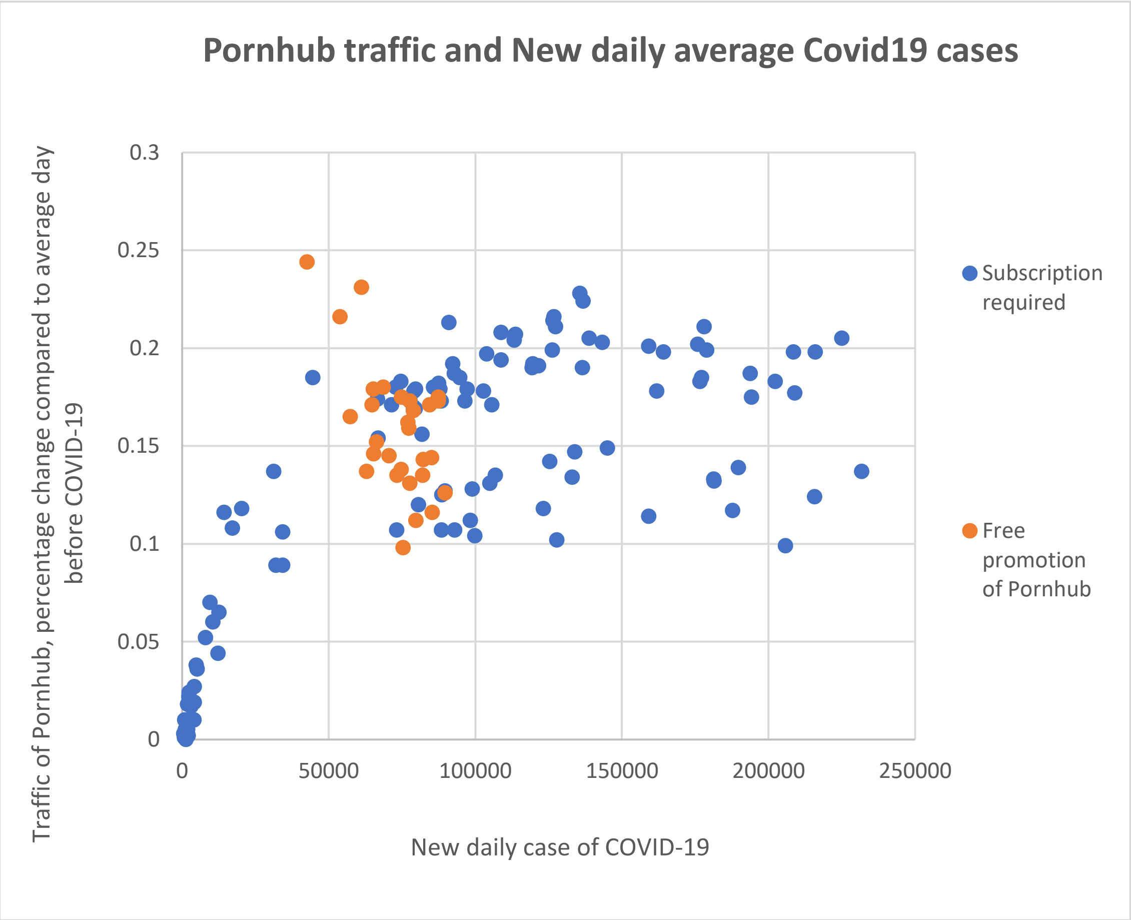

Supplement: S1 Fig — Traffic of Pornhub, percentage change compared to average day before COVID-19. (PDF) [file pone.0260386.s001.pdf]
